# Wrist Extensor Training With Blood Flow Restriction for the Management of Lateral Elbow Tendinopathy: A Case Report

**DOI:** 10.7759/cureus.35468

**Published:** 2023-02-25

**Authors:** Stefanos Karanasios, Ioannis Lignos, Georgios Gioftsos

**Affiliations:** 1 Physiotherapy, University of West Attica, Athens, GRC; 2 Physiotherapy, University of Patras, Patras, GRC

**Keywords:** hypoalgesia, pain threshold, exercise, occlusion training, tennis elbow

## Abstract

Lateral elbow tendinopathy (LET) is a common overuse injury with complex underlying pathophysiological mechanisms. Although several modes of exercise with or without passive interventions have been recommended as the first-line treatment option of the condition, their effectiveness remains inconclusive. The aim of this case report is to evaluate the effect of wrist extensor exercises with blood flow restriction (BFR) as an add-on intervention to a multi-modal physiotherapy programme to improve outcomes in a patient with LET. A 51-year-old male patient presented with a history of right LET for six months. Interventions included wrist extension exercise with BFR, a two-stage progressive loading training programme of the upper limb, soft-tissue massage, education and a home exercise programme for six weeks (12 visits). A substantial improvement in pain intensity, pain-free grip strength, Patient Rated Tennis Elbow Evaluation score and self-perceived recovery was reported at three-, six-, and 12-week follow-up measurements. A 21% reduction in pressure pain thresholds at the lateral epicondyle was found immediately after wrist extensor exercise with BFR. Based on our findings, adding wrist extensor exercises with BFR to a multimodal physiotherapy programme seems a promising approach to improve the treatment outcome in LET. Nonetheless, further research is needed to confirm the present results.

## Introduction

Lateral elbow tendinopathy (LET) or tennis elbow is a frequent musculoskeletal condition that describes a tendinopathy of the common wrist extensors origin [1,2]. The prevalence of LET can reach up to 3% in the general population, 50% in tennis players and 29% in manual workers, respectively [2,3]. Patients with LET present with pain over the lateral epicondyle during gripping and other forceful activities that result in significant disability and increased psychological distress [3]. Several risk factors have been associated with the condition including repetitive use of the tendons, degeneration, smoking, concomitant shoulder tendinopathy, etc. [3].

Despite the recent advances in research regarding the management of patients with LET, their treatment remains difficult possibly due to the complex underlying pathophysiological mechanisms [4,5]. Various non-surgical therapeutic approaches are usually recommended as first-line treatment such as exercises, manual therapy, injections, acupuncture, shockwave therapy, and laser; however, there is no consensus on the optimal therapeutic approach of the condition [6]. Supervised exercises provide a key intervention to improve the treatment outcomes in LET [6]. Nevertheless, contemporary evidence suggests that there is a small clinical benefit in favor of exercise interventions in reducing pain and improving function when compared to other passive interventions or a wait-to-see policy [6,7]. 

Recently, the addition of a novel exercise method using low-load resistance training with blood flow restriction (LLRT-BFR) for the upper limb has been proposed to improve the treatment outcome compared to conventional training in patients with LET [8]. Despite the clinical improvements in function and self-perceived recovery in favor of LLRT-BFR, the trial did not investigate the application of the BFR best practice guidelines for wrist extensors which may explain the poor outcomes regarding pain reduction [8]. Considering that LLRT-BFR has shown significant reductions in pain sensitivity both in healthy individuals and patients with knee pain, we hypothesized that using a programme of wrist extensor training with BFR can reduce pain sensitivity and improve function in patients with LET [9,10].

Therefore, the aim of the present case report was to look at the effect of adding wrist extensor training with BFR in a progressive loading exercise programme for the management of chronic LET. The results of the present report will serve as a pilot study for a future clinical trial of the method in patients with LET.

## Case presentation

History and clinical examination

A 51-year-old businessman (height 175cm; weight 81 kg) presented to our clinic with pain over the lateral side of his right elbow (dominant side). The patient is a recreational tennis player for more than 10 years playing two or three times per week. The patient described a pin-point pain showing over the lateral epicondyle that sometimes may radiate distally to the forearm during strenuous activities. The symptoms started six months ago after a hard tennis game. The pain was easily provoked every day during gripping, after 30 minutes to one hour of keyboard typing, when lifting objects like a cup of coffee or a bag etc. Immediately after the onset of the symptoms, the patient visited an orthopaedic surgeon who prescribed non-steroid anti-inflammatory drugs for a week and a home-based exercise programme for a month that did not help. During the patient’s first visit to our clinic, he reported no symptoms during sleeping or in other sites of the body (cervical spine, shoulder, arm or the non-affected upper-limb etc.). The patient reported not receiving any medication; no previous trauma or history of elbow tendinopathy; no sensory changes. Review of systems was negative for prior surgery, cancer, diabetes, rheumatoid arthritis or neurological problems.

In terms of the clinical examination findings, there were no abnormal findings in posture and gait observation. Also, there were no muscle wasting or inflammatory signs when observing the joints, muscles, and skin of the upper-limbs. Active and passive movements of the neck and shoulders were tested resulting in full range of motion (ROM) and no pain. Assessment of the passive and active movements of the right elbow flexion, extension and pronation were without restriction and pain-free. There was full passive ROM of wrist flexion and extension with the elbow flexed. Active supination and passive wrist flexion with elbow extended provoked patient’s pain over the lateral epicondyle which was self-rated 2 out of 10 in a numerical pain rating scale (NPRS). Active wrist extension with the elbow extended aggravated mostly the patient’s symptom (7 out of 10 in NPRS). The clinical diagnosis of LET was confirmed using the following pain provocation tests: palpation over the lateral epicondyle; resisted wrist extension; resisted middle finger extension; and stretching of the wrist extensors [11].

Outcome measures

During the initial visit, pain intensity was measured on an 11-point numeric pain-rating scale assessing the worst level of pain over the last week [12]. To capture pain and disability we used the Greek version of the Patient Rated Tennis Elbow Evaluation (PRTEE) score [13]. The PRTEE score ranges between 0 and 100, with a score of 100 representing extreme pain and disability [13]. We measured pain-free grip strength (PFGS) using a Jamar hand dynamometer in supine position and the elbow fully extended [14]. We calculated the mean value of three contractions, separated by 30-second rest between repetitions. We presented PFGS measurements as a ratio between the affected and unaffected side [14]. Pain intensity, PRTEE score and PFGS ratio were evaluated at baseline and at the end of the third, sixth and 12th week from the beginning of the rehabilitation programme.

We measured pressure pain thresholds (PPTs) with a 1 cm diameter hand-held digital algometer (Baoshishan ZP-1000 N 20/22806, China) at the lateral epicondyle at both sides [15]. The PPTs were measured five minutes before and after wrist extensor exercise with BFR during the first visit.

We also used a 6-point Likert-scale as a measure of global rating of change (GROC) from “much worse” to “completely recovered” at six- and 12-week follow-ups [16].

Intervention

The patient followed a six-week physiotherapy programme including soft tissue massage, supervised exercises, advice and education. The exercise protocol was based on a previous randomized controlled trial (RCT) that reported significant improvements in improving function and treatment success following an upper-limb training programme with BFR in patients with LET [8]. Despite the positive outcomes, this study did not follow an optimal BFR training protocol for wrist extensors. In the present case report we hypothesized that using explicitly a programme of wrist extensor training with BFR can reduce pain sensitivity and improve function in patients with LET. Therefore, supervised exercises included a two-stage progressive loading programme with the addition of wrist extensor training with BFR (Tables 1, 2). Each session started with four sets (30-15-15-15 repetitions) of wrist extension concentric-eccentric exercise with BFR at 40% of arterial occlusive pressure (AOP). The load was adjusted according to a pain-monitoring approach with acceptable pain during the exercise less than 2 out of 10 in NRPS that increased 0.5-1 kg each week if no pain reported during or after exercises.

**Table 1:** First stage exercises of the supervised exercise programme

1^st^ stage supervised exercises	
1^st^ exercise	Wrist extension with Blood Flow Restriction, 40% of arterial occlusive pressure (30-15-15-15 repetitions), using dumbbells.
2^nd^ exercise	Wrist flexion (3 sets of 10) using dumbbells.
3^rd^ exercise	Supination-pronation (3 sets of 10) using dumbbells. Elbow flexed.
4^th^ exercise	Elbow flexion (3 sets of 10) using dumbbells. Standing position.
5^th^ exercise	Elbow extension (3 sets of 10) using dumbbells. Standing position.
6^th^ exercise	Static stretching exercises of wrist extensors and flexors (3 times x 30s)
Each exercise was performed with a pace of 2 seconds concentric and 2 seconds eccentric phase using a metronome. A 30-second break was used between sets. Load was increased weekly by 0.5-1 kg using a pain-monitoring approach (acceptable pain during the exercise <2 out of 10)	

**Table 2:** Second stage exercises of the supervised exercise programme

2^nd^ stage supervised exercises (After 2 weeks of training in addition to 1^st^ stage exercises)	
7^th^ exercise	Wall push-ups (3 sets of 10)
8^th^ exercise	Wrist extension-flexion using a rubber bar, (3 sets of 10) using dumbbells. Standing position.
9^th^ exercise	Hand grip using a soft ball (3 sets of 10). Standing position.
10^th^ exercise	Standing row with a TheraBand (3 sets of 10)
Each exercise was performed with a pace of 2 seconds concentric and 2 seconds eccentric phase using a metronome. A 30-second break was used between sets. Load was increased weekly by 0.5-1 kg using a pain-monitoring approach (acceptable pain during the exercise <2 out of 10)	

At the first stage the patient performed wrist flexion, elbow flexion, elbow extension and supination-pronation resistance exercises. At the second stage, more exercises were added such as wall push-up, wrist extension-flexion using a rubber bar, handgrip with a soft ball and rowing with a TheraBand. Each session ended with static stretching of the wrist flexors and extensors (three repetitions X 30 seconds) (Tables 1, 2).

Results

There was a substantial reduction (42%) in pain intensity from baseline to three-week follow-up and total absence of pain at six- and 12-week follow-ups (Table 2). Disability decreased from 46% to 26% in PRTEE score after the first three weeks of rehabilitation and resulted in 0 at 12-week follow-up (Table 3). A PFGS ratio of 0.73 was recorded at baseline reaching at 0.95 and 1.03 at six- and 12-week follow-ups, respectively. GROC was rated as ‘much better’ and ‘complete recovery’ at six- and 12-week follow-ups, respectively (Table 3). There was a substantial reduction (21%) in PPTs between before and after wrist extension exercise with BFR at the dominant lateral epicondyle without similar changes at the non-dominant lateral epicondyle (Table 4). The patient reported full adherence to the home-exercise programme while no adverse events or co-interventions were reported.

**Table 3:** Pain intensity, disability score, pain free grip strength ratio and global rating of change at baseline and follow-up measurements. PRTEE, Patients Rated Tennis Elbow Evaluation; PFGS, pain free grip strength  *Expressed as a ratio between the affected and unaffected side

Characteristics	Pain Intensity	PRTEE score	PFGS ratio*	Global rating of change
Baseline	7/10	48/100	0.73	-
Week 3	4/10	26/100	0.81	-
Week 6	0/10	10/100	0.95	‘Much better’
Week 12	0/10	0/100	1.03	‘Complete recovery’

**Table 4:** Pressure pain thresholds (kg/cm2) before and after wrist extensor exercises with blood flow restriction at the affected and non-affected side.

	Pre-intervention	Post-intervention
Dominant Lateral epicondyle	7.5	9.5
Non-Dominant Lateral epicondyle	8.4	8.7

## Discussion

The present report monitored the effect of wrist extension exercises with BFR as an add-on intervention to a six-week supervised training programme in chronic LET. Based on our findings, there were significant improvements in pain, function and self-perceived recovery at six- and 12-week follow-ups. Also, a substantial reduction in pain sensitivity was demonstrated immediately after the wrist extension exercise with BFR at the lateral epicondyle. Our results were in agreement with a previous RCT that suggested better treatment outcomes in favor of upper-limb exercises with BFR to a training programme with progressive loading in LET [8]. Notably, the present report was the first to use a wrist extensor BFR protocol reporting positive outcomes without adverse effects or symptom flare up due to the increased work volume.

Based on multiple research reports LLRT-BFR can improve muscle strength and function in patients with musculoskeletal pathologies; however, in terms of reducing pain intensity research evidence remains inconclusive [17-21]. Specifically, LLRT-BFR does not seem to provide further pain reduction compared to conventional resistance training in patients with knee osteoarthritis [17,20]. Nevertheless, LLRT-BFR shows significantly less knee joint pain compared to non-BFR training in patients after anterior cruciate ligament reconstruction [22]. Possibly, several characteristics such as the type of the problem, the severity and duration of symptoms, or the method of BFR training may influence the results in pain intensity [21]. Currently, the available research trials investigating the changes in pain thresholds after LLRT-BFR have included only healthy subjects suggesting contradictory results [9,23,24]. Although our results indicated a substantial reduction in pain threshold immediately after wrist extension exercise with BFR, further RCTs are required to evaluate the possible hypoalgesic effect of BFR exercises in patients with LET.

It seems that according to the available research evidence selecting the appropriate exercise programme during the management of LET is a challenging procedure. A critical factor for this problem is the paucity of research information regarding the parameters of the proposed training programmes i.e., progression of loading, dosage, volume, time under tension, breaks, acceptable pain level, equipment, frequency and duration. Hence, several modes of exercises have been used in the rehabilitation of patients with LET resulting in conflicting findings [7]. Eccentric training has been proposed as the most effective loading regime especially in the chronic stages of the condition; however, evidence suggests no clinically better outcomes when compared to other types of exercise [7,25]. Similarly, although isometric contractions are usually recommended to reduce pain and improve grip strength in LET at least in the short term, no additional therapeutic benefits have been found compared to a wait-to-see approach in the long term [26]. From another perspective, based on a single study adding shoulder and scapular strengthening exercises to elbow exercises can enhance the clinical outcomes in patients with LET compared to elbow exercises alone [27]. Considering all the previous issues, we developed a detailed progressive loading training programme based on the available evidence that was clinically effective for the management of a patient with chronic LET (Tables 1, 2). Despite the positive findings, the present results should be considered with caution due to the nature of the study design as a case report. Certainly, future randomized clinical trials are needed to evaluate the effectiveness of the proposed training programme compared to other approaches or a control group in patients with LET.

## Conclusions

LET is a common musculoskeletal condition that affects many individuals resulting in a significant functional deficit. Although a multimodal approach including supervised progressive loading exercises is usually recommended, research evidence does not support an optimal training programme for the management of the condition. Based on our findings, the addition of wrist extensor training with BFR to a supervised progressive loading exercise programme along with massage, a home exercise programme and education was effective to reduce pain, improve PFGS and increase function at all follow-up measurements in a patient with LET. Future research using well-designed RCTs is necessary to evaluate the results of the present case report.
